# Effect of Mg Doping on the Physical Properties of Fe_2_O_3_ Thin Films for Photocatalytic Devices

**DOI:** 10.3390/nano12071179

**Published:** 2022-04-01

**Authors:** Rihab Ben Ayed, Mejda Ajili, Yolanda Piñeiro, Badriyah Alhalaili, José Rivas, Ruxandra Vidu, Salah Kouass, Najoua Kamoun Turki

**Affiliations:** 1 LR99ES13 Laboratoire de Physique de la Matière Condensée (LPMC), Département de Physique, Faculté des Sciences de Tunis, Université Tunis El Manar, Tunis 2092, Tunisia; rihab.benayed@fst.utm.tn (R.B.A.); ajili.mejda@yahoo.fr (M.A.); n.najouakamoun@gmail.com (N.K.T.); 2 Nanomag Laboratory, Applied Physics Department, Universidade de Santiago de Compostela, 15782 Santiago de Compostela, Spain; yolanda.fayoly@gmail.com (Y.P.); jose.rivas@usc.es (J.R.); 3 Nanotechnology and Advanced Materials Program, Kuwait Institute for Scientific Research, P.O. Box 24885, Safat 13109, Kuwait; bhalailil@kisr.edu.kw; 4 Faculty of Materials Science and Engineering, University Politehnica of Bucharest, 060042 Bucharest, Romania; 5 Department of Electrical and Computer Engineering, University of California Davis, Davis, CA 95616, USA; 6 Laboratoire des Matériaux Utiles, INRAP, Technopôle, Sidi-Thabet, Tunis 2020, Tunisia; koissa2000@yahoo.fr

**Keywords:** thin films, iron oxide, Mg doping, physical properties, photocatalysis

## Abstract

Undoped and Mg-doped (y = [Mg^2+^]/[Fe^3+^] = 1, 2, 3, and 4 at.%) Fe_2_O_3_ thin films were synthesized by a simple spray pyrolysis technique. The thin films were extensively characterized. X-ray diffraction (XRD) and energy-dispersive spectroscopy (EDS) analysis confirmed the successful insertion of Mg in the rhombohedral structure of Fe_2_O_3_. In addition, scanning electronic microscope (SEM) and confocal microscope (CM) images showed a homogenous texture of the film, which was free of defects. The rough surface of the film obtained by spray pyrolysis is an important feature for photocatalysis and gas sensor applications. The direct band gap of the doped Fe_2_O_3_ films obtained for [Mg^2+^]/[Fe^3+^] = 3 at.% was E_dir_ = 2.20 eV, which recommends the Mg-doped iron oxide as an optical window or buffer layer in solar cell devices. The photodegradation performance of Mg-doped Fe_2_O_3_ was assessed by studying the removal of methylene blue (MB) under sunlight irradiation, with an effective removal efficiency of 90% within 180 min. The excellent photodegradation activity was attributed to the strong absorption of Mg-doped Fe_2_O_3_ in the UV and most of the visible light, and to the effective separation of photogenerated charge carriers.

## 1. Introduction

Maintaining a clean environment has become an inspiring research ambition in the environmental-science-related communities. As a global environmental issue, water pollution threats our life. For example, eco-clean technology is urgently needed to deal with dyes that are manufactured, used, and thrown into the water. Photocatalysis draws attention to the need for extensive research and investigations into water purification and generation of renewable energies [1,2,3,4]. The oxide semiconductor photocatalyst has received special attention as a beneficial material with easy-to-control properties. The basic idea of photocatalysis is the activation of the semiconductor through an artificial or a natural source of light, which breaks down the organic compounds and dyes to purify water [3]. Ferric oxide (Fe_2_O_3_), as an environmental green oxide with outstanding physical and chemical properties, has been the subject of a great deal of research [5,6]. Fe_2_O_3_, as a mid-band gap semiconductor, utilizes sunlight effectively in the photocatalysis process as compared to TiO_2_, which is the most researched photocatalyst [7,8,9,10]. Numerous methods were adopted to enhance the photocatalytic activity in metal oxide semiconductors, such as the formation of junctions and doping to prevent the electron-hole (e^−^/h^+^) recombination and to enhance the photocatalysis process, where dopant ions act as charge traps, reducing the recombination of e^−^/h^+^ [11,12,13]. The photocatalytic degradation of rhodamine B (RhB) with sprayed Fe_2_O_3_ films under sunlight illumination was investigated. The efficiency of RhB decomposition was observed to be 73% during 400 min [14]. Jiamprasertboon et al. prepared an α-/Fe_2_O_3_/ZnO and ZnO/α-Fe_2_O_3_ heterojunction using aerosol-assisted chemical vapor deposition (AACVD). The α-Fe2O3/ZnO exhibited the highest photocatalytic activity under UVA light, which was approximately 16 and 2.5 times higher than that of the Fe2O3 and ZnO layers. In contrast, the reverse heterojunction architecture was less active [15]. Furthermore, Fe_2_O_3_ and Zn:Fe_2_O_3_ nanoparticles were prepared via sol-gel with different Zn ratios. The photodegradation analysis of 4 at.% Zn:Fe_2_O_3_ showed 87% of RB dye degraded in 90 min in the UV light compared to 63% with pure Fe_2_O_3_, and a further increase in Zn content decreased the degradation efficiency [16]. Gajendra K. Pradhan et al. found that Pt-doping enhances the catalytic activity, where, via hydrothermal technique, Pt-doped hematite nanorod showed 63% decomposition of congo red and 55% efficiency towards methylene blue under 4 h of reaction in solar light compared to Fe_2_O_3_ [17]. Satheesh et al. elaborated undoped and doped M:Fe_2_O_3_ (M = Cu, Ni, and Co) using co-precipitation method, with the photocatalytic activity evaluated by the Acid Red-27 (AR-27) degradation under visible light irradiation. Cu:Fe_2_O_3_ showed the highest activity, with around 98.05% of AR-27 being degraded in 90 min, which was higher than that of Fe_2_O_3_ (77.68%), Ni:Fe_2_O_3_ (41.60%), and Co:Fe_2_O_3_ (59.67%) [18]. Nevertheless, an efficient dopant could significantly improve the activity of Fe_2_O_3_. Recently, J. A. Joseph et al. published a paper about Mg-doped iron oxide photocatalytic efficiency. Undoped and doped Fe_2_O_3_ nanostructures were grown by a two-stage electrochemical method. A Photocatalytic activity of 81% was obtained for the Mg:Fe_2_O_3_ nanostructures in 180 min, while Fe_2_O_3_ possessed only 56% of degradation. However, no reports are available on the sprayed Mg-doped hematite for MB degradation [19].

Undoped and doped Fe_2_O_3_ thin films are commonly fabricated by various chemical and physical techniques, such as sol-gel [20], chemical bath deposition [21], chemical spray pyrolysis [22], electrodeposition [23], and SILAR [24]. In addition, low-cost thin films can be deposited on different substrates by chemical spray pyrolysis for various industrial applications [25,26]. In this paper, the structural, morphological, elemental, and optical properties of Mg-doped Fe_2_O_3_ obtained by chemical spray pyrolysis technique are presented. The photocatalytic activity of Mg-doped Fe_2_O_3_ on the photodegradation of methylene blue under sunlight irradiation was investigated, which, to our knowledge, has not yet been studied in the literature.

## 2. Materials and Methods

### 2.1. Preparation of Undoped and Mg-Doped Fe_2_O_3_ Thin Films

Undoped and Mg-doped Fe_2_O_3_ thin films were grown on ordinary glass substrates through chemical spray pyrolysis technique (CSP)**.** Before the deposition process, all the glass substrates were carefully cleaned via immersion in an ultrasonic bath. Iron III chloride (FeCl_3_ and 6H_2_O) and (MgCl_2_ and 6H_2_O) precursors were acquired from AppliChem (Council Bluffs, IA, USA). Both precursors was dissolved in 100 mL of bi-distilled water, where MgCl_2_ was added as a dopant. The Mg ratio was adjusted at y = [Mg^2+^]/[Fe^3+^] = 1, 2, 3, and 4 at.%. On the other hand, the iron chloride concentration was kept at 0.14 mol·L^−1^. The as-prepared solutions were mixed until obtaining homogenous mixtures. The aqueous solution was sprayed with a flow rate equal to 5 mL·min^−1^ by means of compressed air on preheated substrates located 25 cm from the nozzle at 400 °C for 20 min.

### 2.2. Photocatalytic Activity

The photocatalytic activity was assessed by evaluating the degradation of methylene blue (MB) solution under sunlight illumination. The Mg-doped Fe_2_O_3_ film was immersed in 50 mL aqueous MB solution with a concentration of 5 mg/mL and kept under sunlight irradiation for various times (0–180 min). The photodegradation of MB was then estimated by the maximum absorbance at a wave length of 664 nm using Vis-spectrophotometer (Perkin Elmer Lambda 950). According to the Beer–Lambert law, the degradation efficiency of methyl blue was computed starting from the absorbance spectra using the following equation [27]:

(1) $$\mathrm{Degradation}\;\mathrm{Efficiency}\;\left(\%\right)=\frac{\left(A_{0}-A\right)}{A_{0}}\times 100$$

where A_0_ and A are the values of MB solution absorbance at reaction times of 0 and t, respectively.

The reaction kinetic was estimated using the following formula [27]:

(2) $$\mathrm{Ln}\left(\frac{A}{A_{0}}\right)=-\mathrm{kt}$$

### 2.3. Characterization

All the samples were characterized by several techniques as discussed below. The crystalline quality of Fe_2_O_3_ thin layers was characterized by X-ray diffraction (XRD) using X-ray “XPERT-PRO” diffractometer (Malvern Panalytical Ltd., Malvern, UK) with CuKα (λ = 1.54 Å) radiation over a scanning angle (2θ) ranging continuously from 25° to 60°. The experimental XRD spectra were refined with the MAUD software. The surface morphologies and cross-section of the thin films were observed using scanning electronic microscope (SEM) “ZEISS” (Carl Zeiss Microscopy, New York, USA) in surface (1 μm (EHT = 3.00 kV; Mag = 20.00 K) and 200 nm (EHT = 3.00 kV; Mag = 100.00 K)) and cross-section. In addition, 3D characterization was performed using a confocal microscope (CM) (SENSOFAR, Barcelona, Spain). The elemental composition was analyzed using energy-dispersive X-ray spectroscopy (EDS) “ZEISS”. The optical measurements were carried out using a spectrophotometer, Perkin Elmer Lambda 950 (Bridgeport, CT, USA), over the wavelength range of 250–2000 nm.

## 3. Results and Discussion

### 3.1. Structural Properties

**Figure 1:**
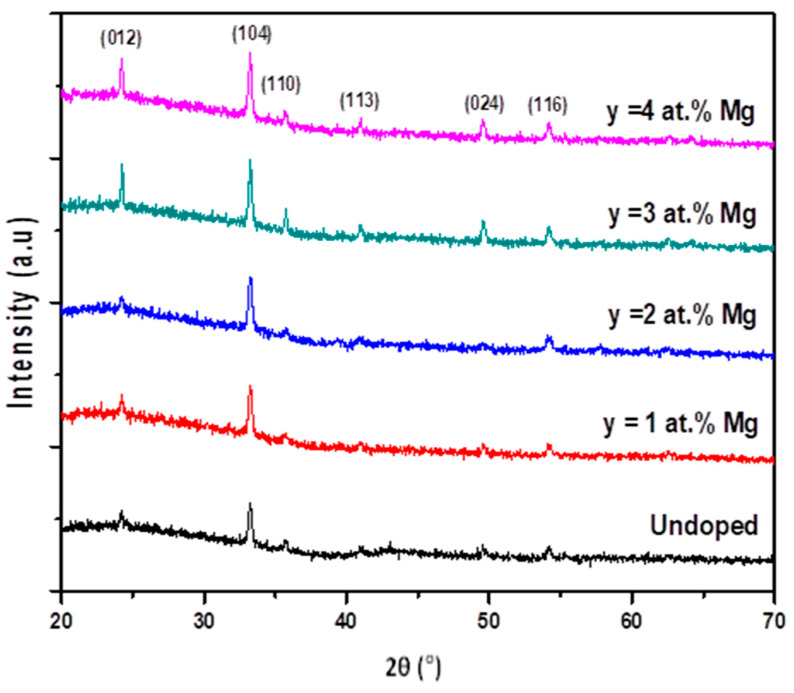
XRD patterns of undoped and Mg-doped Fe_2_O_3_ thin films for different contents (y = [Mg^2+^]/[Fe^3+^] = 1, 2, 3, and 4 at.%).

The influence of Mg-doping on the crystalline structure of the as-synthesized iron oxide composites was examined by X-ray diffraction. As shown in Figure 1, six diffraction peaks located at 2θ = 24.23, 33.24, 35.75, 44.99, 49.56, and 54.18° can be observed in the XRD patterns of the undoped and Mg-doped Fe_2_O_3_ thin films, corresponding to the (012), (104), (110), (113), (024), and (116) planes, respectively, of a typical α-Fe_2_O_3_ phase structure (rhombohedral, R-3C, a = b = 5.023 Å, c = 13.70 Å; JCPDS Card No. 01-089-8104) [28,29]. No new peak formation was observed, which confirms that magnesium has been successfully substituted into the Fe_2_O_3_ matrix. The X-ray analysis shows that all films are polycrystalline and have a preferential orientation along the (104) plane, regardless of the Mg doping level. A slight shift to higher diffraction angles was detected in the XRD scans of the doped samples, especially for 1 at.% and 2 at.%. The shift was attributed to the seamless incorporation of magnesium ions into the Fe_2_O_3_ structure, due to the smaller ionic radius of Mg^2+^ compared to Fe^3+^ [30,31]. We note that the highest intensity of the principal orientation was obtained at 3 at.% Mg doping concentration.

**Figure 2:**
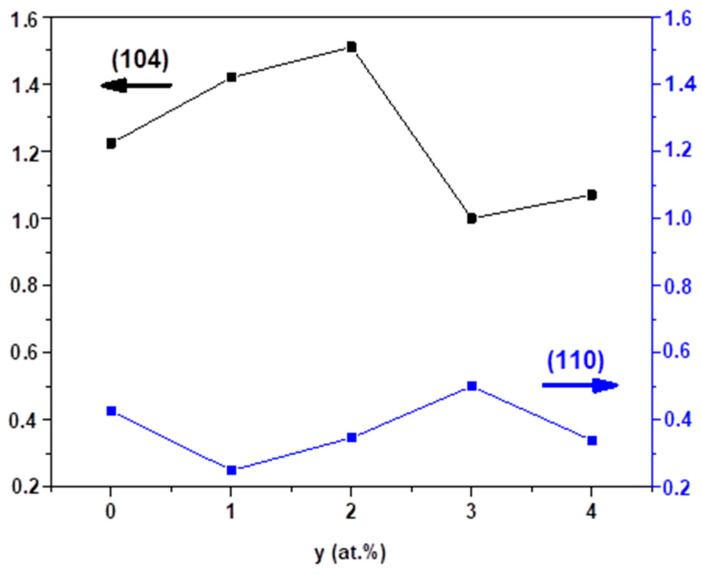
Texture coefficient (T_C_) for (104) and (110) directions in undoped and Mg-doped Fe_2_O_3_ grown films (0 ≤ y = [Mg^2+^]/[Fe^3+^] ≤ 4 at.%).

The preferred orientation degree (Tc) in the (hkl) orientation was determined using the following empirical relation [27]:

(3) $$T_{C_{\left(\mathrm{hkl}\right)}}=\frac{I_{\left(\mathrm{hkl}\right)}/I_{0\left(\mathrm{hkl}\right)}}{\frac{1}{N}\sum_{N}\left(\frac{I_{\left(\mathrm{hkl}\right)}}{I_{0\left(\mathrm{hkl}\right)}\;}\right)}$$

where (hkl) are the Miller indices, I_0_ is the standard intensity, I is the measured intensity, and N is the number of reflection peaks. The variation of the Tc (104) and Tc (110) values with the doping concentration of Mg is represented in Figure 2, which shows that the tendency of crystallites to develop along the (110) plane increases with the increase of the Mg doping concentration up to 3 at.%. Although (104) is the major orientation, the variation of the intensity of the (110) plane, which presents a random orientation of crystallite, confirms the polycrystalline character of the Mg-doped thin films.

**Figure 3:**
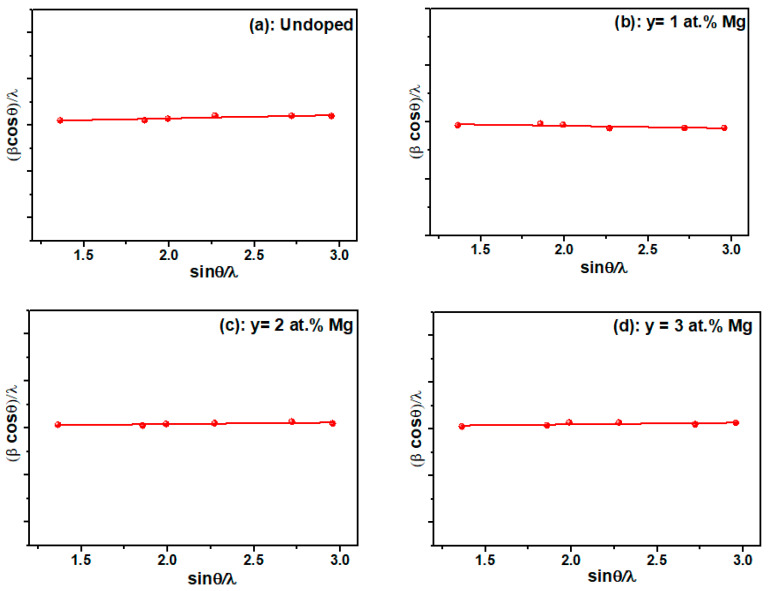

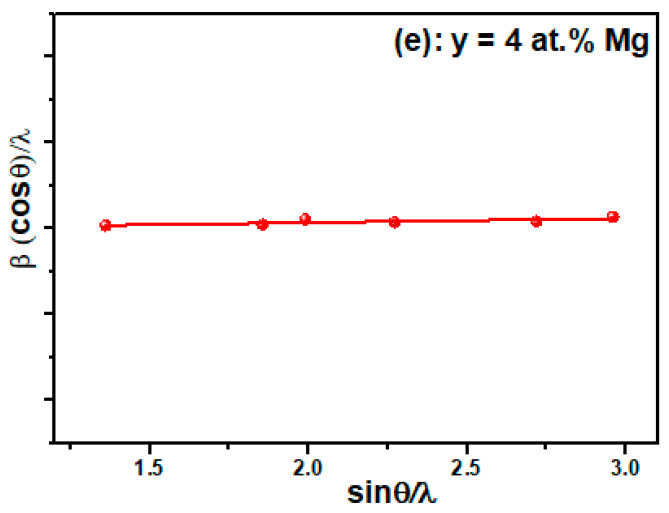
Williamson–Hall plots for the undoped and Mg-doped Fe_2_O_3_ thin films.

The lattice constants a and c of the nanocrystals and the cell volume of all the samples were estimated according to the following equations [7]:

(4) $$\frac{1}{d_{\mathrm{hkl}}^{2}}=\frac{4}{3a^{2}}\left(h^{2}+k^{2}+\mathrm{hk}\right)+\frac{l^{2}}{c^{2}}$$

(5) $$V=\frac{\sqrt{3}}{2}a^{2}c$$

where d_hkl_ is the interplanar distance. The estimated values of lattice parameter are presented in Table 1. The calculated values of the unit cell parameters of Mg-doped Fe_2_O_3_ were found to be lower than those of the undoped films, which result in a decrease in the cell volume. Magnesium incorporation caused local changes in the Fe_2_O_3_ matrix, which confirms the successful fabrication of Mg-doped Fe_2_O_3_ thin film [32,33,34]. For further investigation of the impact of Mg doping on the film microstructure, the average crystallite size and macrostrain of the films were estimated using Williamson–Hall formula [22]:

(6) $$\frac{\beta \cos\theta }{\lambda }=\frac{1}{D}+\varepsilon \frac{\sin\theta }{\lambda }$$

where β is FWHM in radian, D is the grain size in nanometers, ε is the microstrain, and λ is X-ray wavelength in nanometers. The values estimated from Figure 3 are presented in Table 1, which shows that D decreased from 60.8 nm to 55.3 nm regardless of the doping concentration. Consequently, the microstrain increased from 0.31 × 10^−3^ to 0.64 × 10^−3^. The decrease in the lattice parameters (a,c) and cell volume (V) presented in Table 1 is expected due to the stoechiometric replacement of Fe ions with Mg ions with smaller ionic radii. The smallest volume, V, was obtained for y = 3 at.%, corresponding to the best crystalline quality (Figure 1 and Figure 2), which is also in agreement with the substitution of Fe by Mg in the lattice [33].

### 3.2. Rietveld Analysis

**Figure 4:**
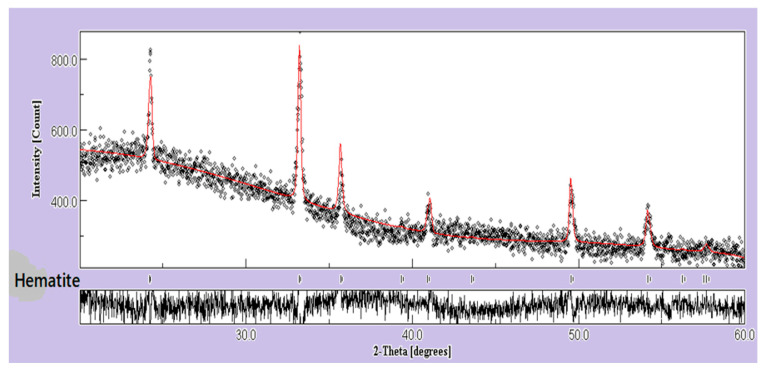
Rietveld refinement patterns of 3 at.% Mg-doped Fe_2_O_3_ samples (dots for experimental and solid line for simulated curves).

Rietveld analysis (using MAUD software) was used for the 3 at.% Mg-doped Fe_2_O_3_ thin films to check the α-Fe_2_O_3_ phase purity. The refinement plot is shown in Figure 4, which represents the experimental pattern as black dots and the pattern calculated with Rietveld refinement as red solid lines. In addition, the lower part of the graph indicates the difference between the values of experimental and calculated intensities. The structural fitting quality was checked by the goodness-of-fit factor (GoF = Rwp/Rexp), where Rwp is the weighted residual error and Rexp is the expected error. The GoF was found to be 1.26, which describes a well-fitting model with low discrepancies between the experimental and calculated XRD patterns [7,35]. The results obtained confirm that the Fe ion was substituted by the Mg ion in the Fe_2_O_3_ phase.

### 3.3. Morphological Properties

**Figure 5:**
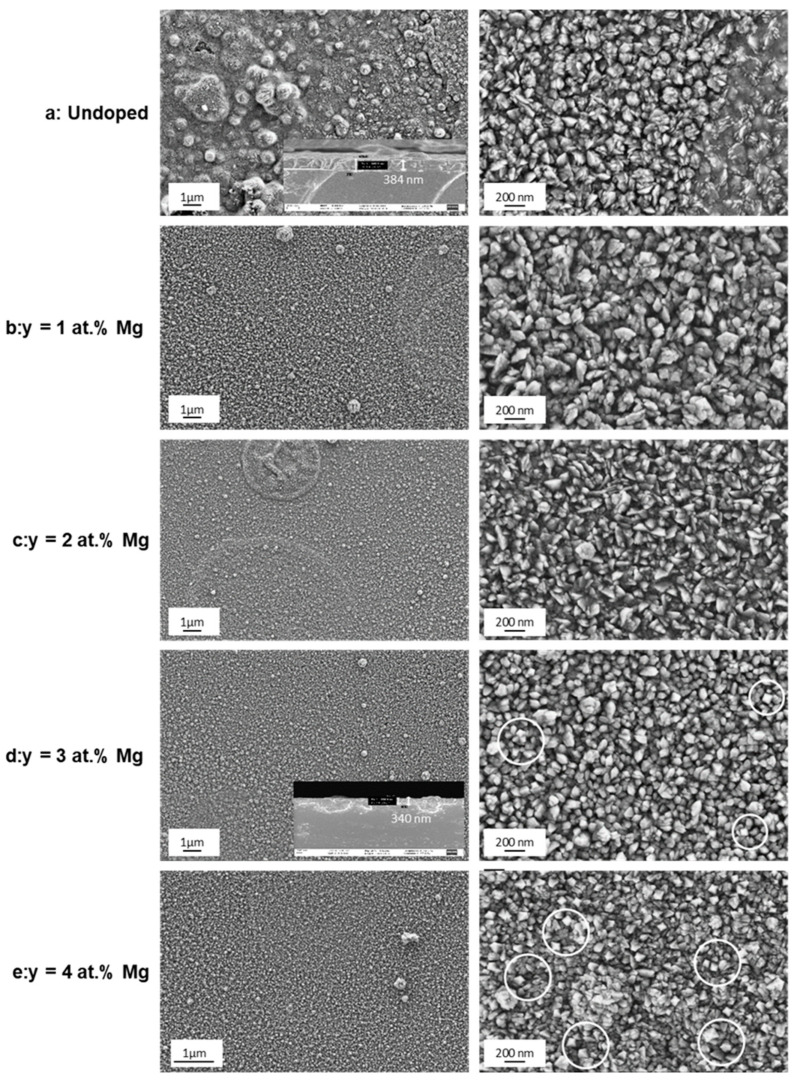
SEM images at two magnifications (1 μm and 200 nm) of the undoped and Mg-doped Fe_2_O_3_. The inlets show the cross-sections of the respective thin films.

The effect of magnesium doping on the morphology of Fe_2_O_3_ films is shown in Figure 5a–e. The SEM micrographs show a granular morphology with a uniform distribution through the substrate surface for the undoped and doped samples. A similar morphology was mentioned in literature [5,22,28]. The cross-section images show a compact and homogenous textured film with a thickness of about 384 and 340 nm for undoped Fe_2_O_3_ and 3 at.% Mg-doped Fe_2_O_3_, respectively (Figure 5a,d). No defects are observed on the samples surface. The undoped films appear to have a randomly distributed grain agglomeration on the surface of the film. The Mg-doped films show a more uniform distribution with smaller grain size compared to the undoped films, which recommends them for photocatalysis applications [36]. At a higher Mg-doping concentration, the rhombohedral shape of the grains appears more clearly defined (inset Figure 5d,e).

**Figure 6:**
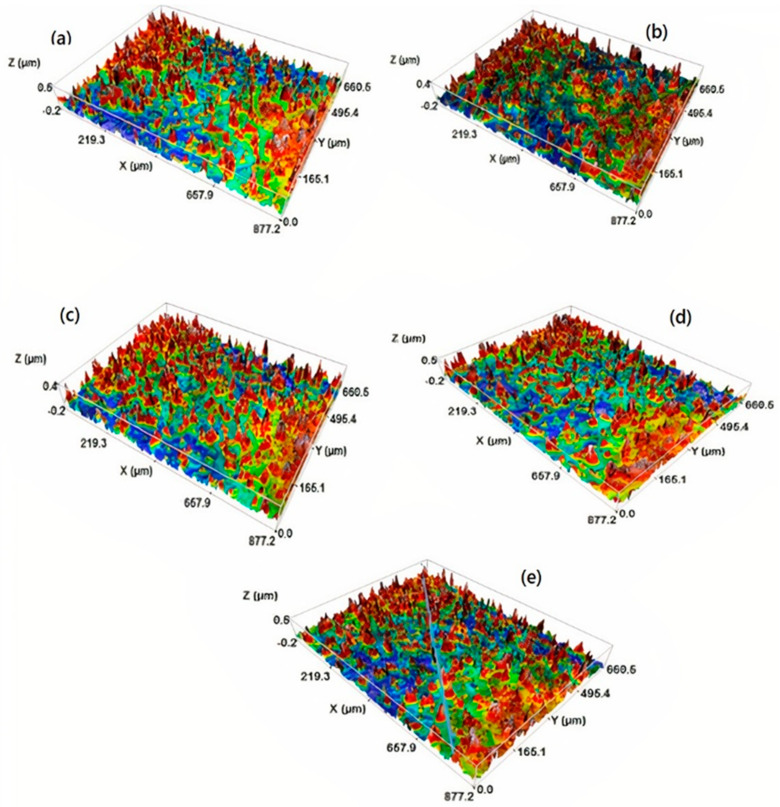
3D CM micrographs of undoped and Mg-doped Fe_2_O_3_ with different contents ((**a**) undoped, (**b**) 1 at.%, (**c**) 2 at.%, (**d**) 3 at.%, and (**e**) 4 at.%).

In order to better understand the effect of Mg doping on the morphological properties of the Fe_2_O_3_ thin films, the topography evolution of the deposited films was investigated using confocal microscope (CM). Figure 6 presents the 3D CM images of the thin films. Overall, these images are in agreement with those observed by SEM (Figure 5). The 3D micrographs show a homogenous layer free from voids and cracks. The surface roughness parameters, including the root mean square (Sq) and arithmetic average of absolute values (Sa), were extracted from CM data. The values of surface roughness of undoped and Mg-doped Fe_2_O_3_ thin films as a function of Mg concentrations are given at Table 2. The Sa and Sq values vary in the range [60.6–76.6] nm and [80.6–104.1] nm, respectively. Mg-doped Fe_2_O_3_ layers have a relatively high surface roughness, which can offer more available active sites and, consequently, improve the adsorption of pollutants during the photocatalysis process [18]. The highest values of Sa and Sq were obtained for a Mg content of 3 at.% (Table 2).

**Figure 7:**
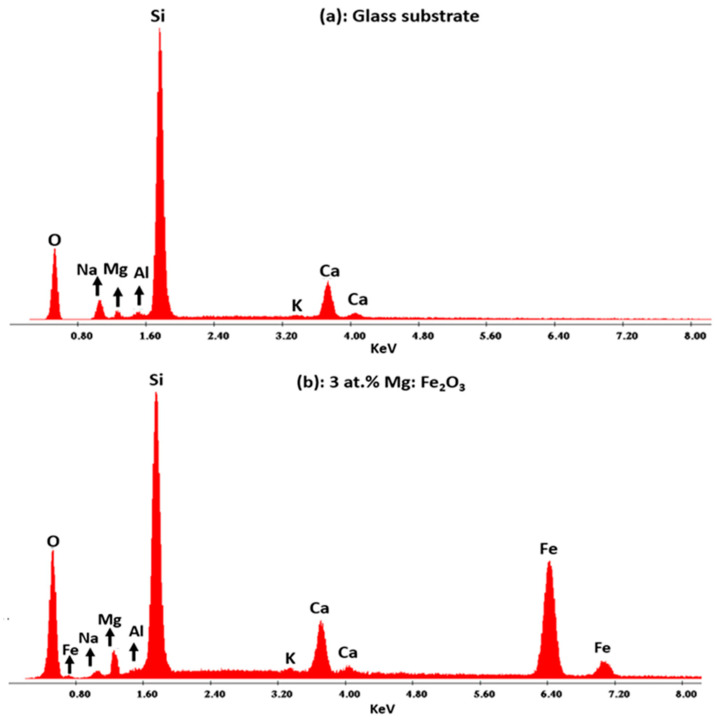
EDS Spectrum of the glass substrate (**a**) and 3 at.% Mg-doped Fe_2_O_3_ film (**b**).

The EDS spectra of the glass substrate and 3 at.% Mg-doped Fe_2_O_3_ film shown in Figure 7 reveal many peaks. The EDS analysis confirmed the presence of the expected elements, iron (Fe), magnesium (Mg), and oxygen (O), all in addition to those attributed to glass substrate, confirming the grown layer free from impurities.

### 3.4. Optical Properties

**Figure 8:**
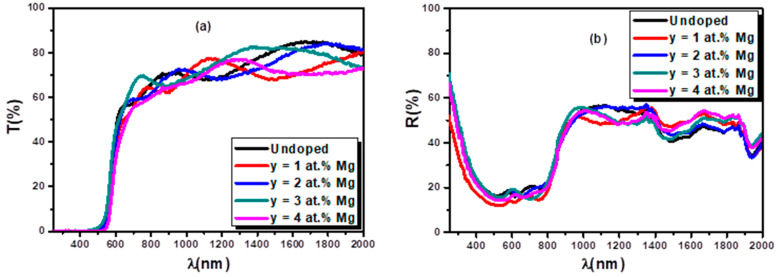
The optical transmittance (T) (**a**) and reflectance (R) (**b**) of undoped and Mg-doped Fe_2_O_3_.

To investigate the doping effect of Mg (y = 1, 2, 3, and 4 at.%) on the optical properties of Fe_2_O_3_ thin films, transmission (T(λ)) and reflection (R(λ)) measurements were carried out. The T (λ) and R(λ) spectra are presented in Figure 8, which shows that the as-deposited films have a high transmission coefficient (≥60%) within the interval of 1000–2500 nm. Below 650 nm, a sharp fall in all the R–T spectra is observed, which is due to the very strong absorption of these films in the UV and most of the visible light region. Remarkably, in the SEM cross- section images (Figure 5), interference fringes are observed in the transmission spectra, referring to the films excellent thickness homogeneity [22,28]. The optical reflection data allow the estimation of the band gap values through the differential reflectance spectra (dR/dλ) as a function of wavelength. The optical direct (Edir) and indirect (Eind) band gaps values obtained for Fe_2_O_3_ and Mg-doped iron oxide semiconductor are typical of those for Fe_2_O_3_ (Table 3) [7]. No significant variation was observed for indirect transition (E_ind_), but a slight increase of the direct transition value (E_dir_) with Mg doping content was observed from 2.15 eV for Fe_2_O_3_ to 2.20 eV for 3 at.% Mg-doped Fe_2_O_3_ (Table 3). We observed that the films crystallinity improved with the Mg insertion (Figure 1). In addition, a slight increase in the direct band gap was noticed, to reach a maximum value equal to 0.05 eV with a magnesium doping ratio equal to 3 at.%. This finding is in good correlation with the crystallinity observed at the same doping ratio, which reduced the structural defects.

### 3.5. Photocatalytic Activity

**Figure 9:**
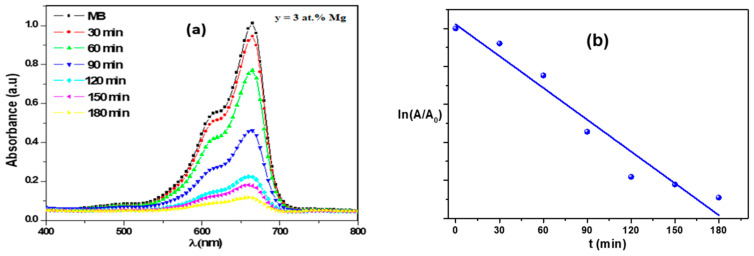
Time-dependent absorption spectra of MB dye solution in the presence of Fe_2_O_3_ (**a**), 3 at.% Mg-doped Fe_2_O_3_ (**b**) and kinetic degradation of MB dye.

It is well recognized that the photocatalysis process occurs through the photogeneration of electron-hole pairs under light irradiation [2,4]. When photons reach a semi-conductor catalyst, an electron absorbs the photon energy to move from valence band to occupy conduction band levels, leaving behind an electron vacancy (hole); thus, creating the electron-hole pairs. On the other hand, the lifetime of electron-hole pairs is very short [13,15,17]. To ensure the conservation of the charges, that is when the electron and/or hole is filled by an arbitrary charge, and the photocatalytic reaction may take place. In order to study the photocatalytic activity of Mg-doped Fe_2_O_3_ thin films, many parameters such as adsorption process, particle size, morphology, and crystallinity catalyst performance were considered. Mg-doped Fe_2_O_3_ with y = 3 at.% provides a good platform for the photodegradation of organic dyes. Figure 9a shows the absorbance spectra recorded in the wavelength range from 400 to 800 nm. As shown in the absorption spectra of MB, the main peak intensity occurred at 664 nm over the reaction time using Mg-doped Fe_2_O_3_ as catalyst. As irradiation was carried out, the absorption intensity decreased, with about 90% of degradation achieved within 180 min (Figure 9b) This film shows higher degradation efficiency compared with the undoped Fe_2_O_3_ thin films, which degraded almost 50% of MB under (Figure 9a) sunlight irradiation. The substitution of Fe^3+^ by Mg^2+^ sited onto the Fe_2_O_3_ lattice led to the enhancement in the photocatalytic activity. The possible reaction mechanism for the photocatalytic process of the mentioned sample is shown below [12]:

Mg:Fe_2_O_3_ + hν → Mg:Fe_2_O_3_ (e^−^ + h^+^) (7)

e^−^_BC_+ O_2_ → O_2_° (8)

h^+^_BV_+ H_2_O → OH° + H^+^ (9)

MB + (OH°, O_2_°^−^) → Intermediates → Decomposition product (10)

As a result, the linked works of the photogenerated electrons and holes cause the MB degradation, where the e- reacts with oxygen molecules to produce superoxide anion radicals (O_2_°^−^) and the h^+^ reacts with water to form hydroxyl radicals. The inserted magnesium ions act like a charge sink, which consequently can enhance the separation of the photogenerated charge pairs, giving rise to further superoxide and hydroxyl radicals, leading to an enhancement in the photocatalytic degradation efficiency. Many researchers have reported that the activity of semiconductor materials in dye degradation processes is affected by metal transition dopants [13,17,18]. In order to investigate the kinetic of MB photodecomposition on the catalyst surface, Figure 9b shows the curve fitting of the kinetic equation. A straight line was obtained, indicating that the reaction is of pseudo-first order. Thus, 3 at.% Mg-doped Fe_2_O_3_ thin layer presents a performant photocatalyst.

## 4. Conclusions

Fe_2_O_3_ and Mg-doped Fe_2_O_3_ films were grown using chemical spray pyrolysis technique and then characterized using several analytical techniques. A structural characterization study revealed that all the films are highly crystallized in the rhombohedral structure with (104) as principal orientation, which confirms that Mg was well-incorporation onto the Fe_2_O_3_ lattice, especially for 3 at.% of Mg-doped Fe_2_O_3_. The morphological properties of the films showed a rough surface with granular texture, with the highest values of Sa and Sq for 3 at.% Mg-doped Fe_2_O_3_. Moreover, the roughness values obtained for 3 at.% Mg-doped Fe_2_O_3_ may improve the sensitivity of iron oxide to detect toxic gases, which recommends the 3 at.% Mg-doped Fe2O3 thin film for gas sensor devices. The Mg doping did not significantly affect the indirect optical band gap (Eind), while the direct band gap increased from 2.15 eV for undoped films to 2.20 eV for 3 at.% Mg-doped Fe_2_O_3_. These band gaps values of Mg-doped Fe_2_O_3_ recommend the use of these films as an optical window or buffer layer in photovoltaic devices. Otherwise, magnesium incorporation enhanced the photogenerated charge separation, greatly improving the photocatalytic performance of iron oxide material. The dye degradation activity of the 3 at.% Mg-doped Fe_2_O_3_ catalyst reached 90% after 180 min of sunlight irradiation.

## Figures and Tables

**Table 1:** The variation of grain size (D), microstrain (ε), and lattice parameters (a and c) and unit cell volume (V) versus Mg (at.%) content.

Mg (at.%)	D (nm)	ε × 10^−3^	a (Å)	c (Å)	V (Å^3^)
0	60.8	0.31	5.034	13.77	302.18
1	59.2	0.53	5.026	13.74	300.57
2	57.7	0.55	5.024	13.73	300.11
3	55.3	0.62	5.022	13.72	299.65
4	55.9	0.64	5.024	13.72	299.89

**Table 2:** The surface arithmetic average of absolute values (S_a_) and root mean square (S_q_) values for undoped and Mg-doped Fe_2_O_3_ thin films.

Mg (at.%)	S_a_ (nm)	S_q_ (nm)
0	70.7	95.1
1	63.4	85.5
2	60.6	80.6
3	76.6	104.1
4	74.9	102.6

**Table 3:** Direct (E_ind_) and indirect (E_dir_) band gaps energies values of undoped and Mg-doped Fe_2_O_3_ thin films.

Mg (at.%)	E_dir_	E_ind_
0	2.15	1.97
1	2.16	1.96
2	2.18	1.96
3	2.20	1.99
4	2.16	1.97

## Data Availability

The data is available on reasonable request from the corresponding author.
